# Development of UHPC Mixtures Utilizing Natural and Industrial Waste Materials as Partial Replacements of Silica Fume and Sand

**DOI:** 10.1155/2014/713531

**Published:** 2014-08-13

**Authors:** Shamsad Ahmad, Ibrahim Hakeem, Mohammed Maslehuddin

**Affiliations:** ^1^Civil and Environmental Engineering Department, King Fahd University of Petroleum and Minerals, Dhahran 31261, Saudi Arabia; ^2^Research Institute, King Fahd University of Petroleum and Minerals, Dhahran 31261, Saudi Arabia

## Abstract

In the exploratory study presented in this paper, an attempt was made to develop different mixtures of ultrahigh performance concrete (UHPC) using various locally available natural and industrial waste materials as partial replacements of silica fume and sand. Materials such as natural pozzolana (NP), fly ash (FA), limestone powder (LSP), cement kiln dust (CKD), and pulverized steel slag (PSS), all of which are abundantly available in Saudi Arabia at little or no cost, were employed in the development of the UHPC mixtures. A base mixture of UHPC without replacement of silica fume or sand was selected and a total of 24 trial mixtures of UHPC were prepared using different percentages of NP, FA, LSP, CKD, and PSS, partially replacing the silica fume and sand. Flow and 28-d compressive strength of each UHPC mixture were determined to finally select those mixtures, which satisfied the minimum flow and strength criteria of UHPC. The test results showed that the utilization of NP, FA, LSP, CKD, and PSS in production of UHPC is possible with acceptable flow and strength. A total of 10 UHPC mixtures were identified with flow and strength equal to or more than the minimum required.

## 1. Introduction

UHPC, also known as reactive powder concrete (RPC), exhibits excellent mechanical and durability properties and is one of the latest advances in concrete technology. The high compressive strength (more than 150 MPa), tensile strength, toughness, and ductility along with negligible water and chloride permeability, and therefore high durability, of this new concrete material make it UHPC [1]. The basic principle on which UHPC is based is to achieve a cement matrix as dense as possible (by reducing microcracks and capillary pores in the cement matrix) and a dense transition zone between cement matrix and aggregate. These requirements of UHPC are achieved by enhancing the homogeneity by replacing coarse aggregate by fine quartz sand with a maximum size of 600 *μ*m [2]; improving the properties of cement matrix by the addition of pozzolanic admixture, such as silica fume in the range of 15% to 30% of the mass of cement [2, 3]; reducing water to binder ratio to below 0.2 (by mass) with the help of a high dosage of superplasticizer; optimizing the particles grading to achieve maximum packing density of mixture; adding an adequate amount of steel fibers to achieve ductility; and adopting a suitable method of curing [4–7].

As a result of extensive research carried out globally during the last few years, the production of UHPC is no longer limited within the domain of patented concrete materials. However, use of a very high amount of silica fume and the requirement of fine quartz sand in UHPC put bottlenecks in producing UHPC in places where such ingredients are locally unavailable. In order to mitigate this problem, the possibility of using locally available alternative materials as partial replacement for silica fume and fine quartz sand should be explored. Several studies are reported on production of UHPC utilizing different mineral admixtures [8–11].

Through a study on use of pulverized fly ash, pulverized granulated blast furnace slag, and silica fume as a partial replacement of cement, Yazıcı [8] has found that high strength concrete with compressive strength more than 170 MPa can be produced. Basalt and quartz powder were used as an aggregate in the mixtures and three different curing methods (standard, autoclave, and steam curing) were applied to the specimens.

Yazici et al. [9] have reported the effect of partial replacement of the cement and silica fume (SF) by fly ash (FA) and/or ground granulated blast furnace slag (GGBFS) on the performance of RPC. Their test results indicated that the utilization of FA and/or GGBFS in RPC is possible without significant loss of mechanical performance. They concluded that the RPC containing high volume binary (SF-FA or SF-GGBFS) or ternary (SF-FA-GGBFS) blends have satisfactory mechanical performance. In other words, utilization of FA and/or GGBFS in RPC production is very effective.

In another study, Yazıcı et al. [10] have investigated the mechanical properties (compressive strength, flexural strength, and toughness) of RPC produced with class-C FA and GGBFS under different curing conditions (standard, autoclave, and steam curing). They have observed that by increasing the GGBFS and/or FA content, the toughness of RPC increases under all curing regimes considerably. Furthermore, SEM micrographs revealed dense microstructure of RPC. The test results also showed that RPC containing high volume mineral admixtures has satisfactory mechanical performance. Although the cement and silica fume contents of these mixtures were lower than the conventional RPC, compressive strength exceeded 200 MPa after standard water curing. Finally, they reported that the GGBFS and/or FA can also be used as a fine silica source for RPC.

Van Tuan et al. [11] investigated the possibility of using rice husk ash (RHA) to produce UHPC. RHA is an agricultural waste which possesses a very high amount of amorphous SiO_2_ and a large surface area and is therefore classified as a “highly active pozzolana.” The result showed that the compressive strength of UHPC incorporating RHA, can be achieved in excess of 150 MPa with normal curing regime. The interesting point is that the effect of RHA on the development of compressive strength of UHPC is larger than that of SF. Besides, the sample incorporating the ternary blend of cement with 10% RHA and 10% SF showed better compressive strength than that of the control sample without RHA or SF. This blend proved to be the optimum combination for achieving maximum synergic effect.

Taking notes of the research work pertaining to the utilization of various natural and waste materials in producing UHPC, an attempt was made under the present work to develop alternative mixtures of UHPC using NP, FA, LSP, CKD, and PSS, the materials locally available in Saudi Arabia. The main objective of this study was to explore the possibility of reducing the consumption of silica fume through its replacement by NP, FA, LSP, CKD, and PSS without compromising with the required flow and compressive strength.

## 2. Experimental Program

The experimental program consisted of first selecting different viable percentages of NP, FA, LSP, CKD, and PSS for replacing microsilica and dune sand contents of a typical base mixture of UHPC possessing a flow of 230 mm and 28-d compressive strength of 161 MPa. Using permutations and combinations of the replacements of microsilica and dune sand, a set of 24 UHPC trial mixtures were designed using absolute volume method. These mixtures were prepared and tested for flow before casting to obtain specimens for compressive strength testing after 28 days of water curing.

### 2.1. Materials

#### 2.1.1. Cement

Ordinary Portland cement conforming to ASTM C150 type I with a specific gravity of 3.15 was used in all the UHPC mixtures. Sufficient amount of cement was procured and stockpiled safely to prevent hardening of cement. The chemical composition of cement is shown in Table 1.

#### 2.1.2. Aggregate

Dune sand, abundantly available in the deserts of Saudi Arabia, was used in this study as aggregate in its naturally graded form.  Table 2 shows the grading of the dune sand used. The specific gravity of fine aggregate was 2.53, and the water absorption was 0.4%.

#### 2.1.3. Superplasticizer

The superplasticizer used in all the trial mixtures was Glenium 51. It is a new generation polycarboxylic-based ether hyperplasticiser. It was sourced from a local supplier in Saudi Arabia. Its technical data, as obtained from the manufacturer, is shown in Table 3.

#### 2.1.4. Microsilica (MS)

Elkem microsilica, generated from the carbothermic reduction of quartz and quartzite in electric arc furnaces in the production of silicon and ferrosilicon alloys, containing 85–95% SiO_2_ with very fine vitreous particles (fineness in the order of 10 times finer than that of cement), was used in this study. The chemical composition of the microsilica used in this study is shown in Table 4. The microsilica had a specific gravity of 2.25.

### 2.2. Natural and Industrial Waste Materials

Several natural and industrial waste materials are abundantly available in Saudi Arabia at relatively lower costs, which can be used as replacing materials in producing UHPC concrete mixtures. Details of five such materials are presented in Table 5. These materials were used in varying percentages as partial replacements of microsilica and sand. It can be observed from Table 5 that while NP and FA are rich in silica, LSP, CKD, and PSS are rich in lime. Considering this fact, maximum replacement of microsilica by NP and FA was kept up to 80% whereas the replacement of microsilica by LSP, CKD, and PSS was limited to a maximum level of 20%.

### 2.3. Base Mixture of UHPC

A typical mixture of UHPC developed earlier by the authors with a flow of 230 mm and 28-d compressive strength of 161 MPa was considered as a base mixture without replacement of microsilica and sand by the natural and industrial waste materials. The quantities of constituent materials for producing 1 m^3^ of the selected base UHPC mixture are shown in Table 6. As can be observed from Table 6, base mixture contains the following: cement forming about 36.2% weight of the mixture, fine dune sand forming about 40.5% by weight of the mixture, Elkem microsilica forming about 8.9% by weight of the mixture, a water-to-binder ratio of about 0.145 (by weight), the superplasticizer (Glenium 51) forming about 1.6% by weight of the mixture (3.5% by mass of binder), water forming about 6.5% by weight of mixture, and steel fibers (with diameter of about 0.15 mm, length of about 12.7 mm, and tensile strength over 2500 MPa) forming about 6.3% by weight of the mixture.

### 2.4. Trial Mixtures of UHPC Using Natural and Industrial Waste Materials

For preparing trial mixtures of UHPC, microsilica (out of 220 kg/m^3^ used in base mixture) was partially replaced by NP and FA in the range of 40%, 60%, and 80% and by LSP, CKD, and PSS in the range of 5%, 10%, and 20%. Dune sand (out of 1005 kg/m^3^ used in base mixture) was partially replaced by LSP, CKD, and PSS in the range of 5%, 10%, and 20%. This way a total of 24 trial mixtures of UHPC were considered. The design of all these trial mixtures was carried out using absolute volume method. The water/binder ratio and quantities of water, cement, superplasticizer, and steel fiber were kept constant at values the same as those for the base mixture of UHPC. The estimated quantities of all ingredients for producing 1 m^3^ of the trial mixtures are presented in Table 7 along with the ID of each of the UHPC mixtures.

### 2.5. Preparation and Testing of Trial Mixtures of UHPC

For preparing the UHPC trial mixtures, batching of all ingredients was done as per their quantities listed in Table 7 and a step-by-step procedure for charging and mixing was adopted, as outlined below.

- Cement, silica fume, and dune sand were charged together in a Hobart planetary high speed mixer and allowed to get mixed slowly for a duration of 2 minutes.
- Half of the total quantity of superplasticizer was mixed with water and the mixture of water and superplasticizer was added slowly to the dry mixture of cement, silica fume, and dune sand. The mixing was continued for 8 to 10 minutes until the dry mixture is converted into granules.
- After formation of the granules, the remaining half of the superplasticizer was added slowly and mixing was continued for about another 5 minutes until the mixture was turned into a homogenous fluid.
- Finally, the steel fibers were added to the mixture slowly in small amounts over the course of the next 2 minutes. After the fibers were charged completely, the mixing was continued for a further period of 3 minutes to ensure that the fibers were well dispersed in the prepared mixture of UHPC.
- The prepared mixture of UHPC was then taken for first conducting flow test and then casting the specimens for compressive strength. It should be noted that the mixing times in each step are relative and are only specifically applicable to the mixer used in this study.

ASTM C1437 standard test method for measuring flow of hydraulic cement mortar was used to determine flow of the trial mixtures of UHPC. For measuring flow, a minislump cone was filled with the UHPC mixture and then removed slowly to allow the mixture to flow evenly on the table and then the flow table was lifted up and dropped down for 20 times to allow the mixture to spread on the flow table. The average diameter of the spread mixture was recorded as flow value for the mixture. The acceptable value of mixture flow ranges between 180 mm and 220 mm. The flow test was completed and mixture was cast within first 20 minutes of the mixing to obtain specimens for compressive strength test. The casting of the specimens for compressive strength test was done by pouring the mixture into moulds kept on a vibrating table and then vibrating the table for about 30 seconds after filling to consolidate the mixture. After casting, the specimens were covered with plastic sheet for 24 hours in the laboratory environment (22 ± 3°C) and then submerged in water tank for 28-d curing before testing for compressive strength.

## 3. Results and Discussion

**Figure 1:**
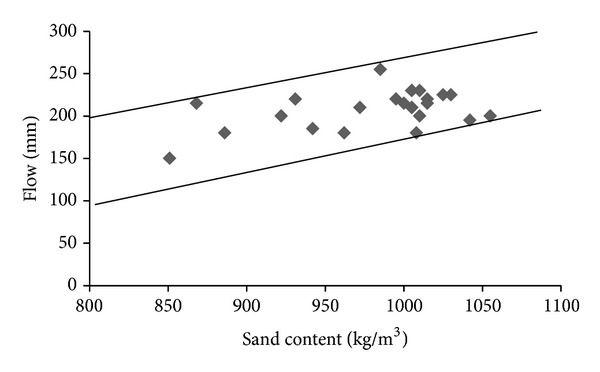
Variation of flow with sand content.

**Figure 2:**
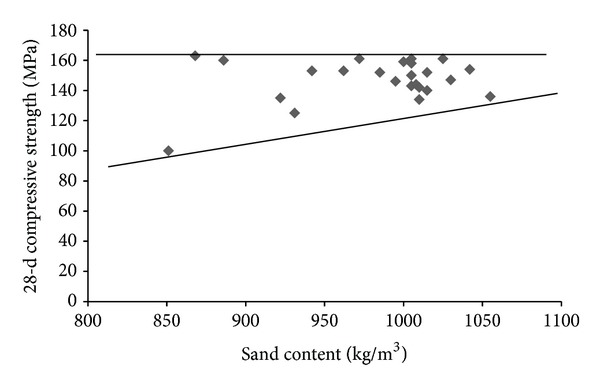
Variation of 28-d compressive strength with sand content.

The flow and 28-d compressive strength test results for all trial mixtures and base mixture are presented in Table 8. As can be observed from Table 8, the flow and 28-d compressive strength of trial UHPC mixtures varied in a wider range of 150 to 255 mm and 125 to 163 MPa, respectively. As can be seen from Table 7, the sand content of the trial mixtures varies in a wider range of 764 to 1055 kg/m^3^ due to replacement of microsilica and dune sand by the replacing materials. To observe the effect of variation of sand content on flow and compressive strength, Figures 1 and 2 were plotted using the data from Table 8. It can be observed from Figure 1 that the flow of the mixtures is slightly improved with increase in the sand content.  Figure 2 indicates that there is no clear trend of variation of compressive strength with change in the sand content.

**Figure 3:**
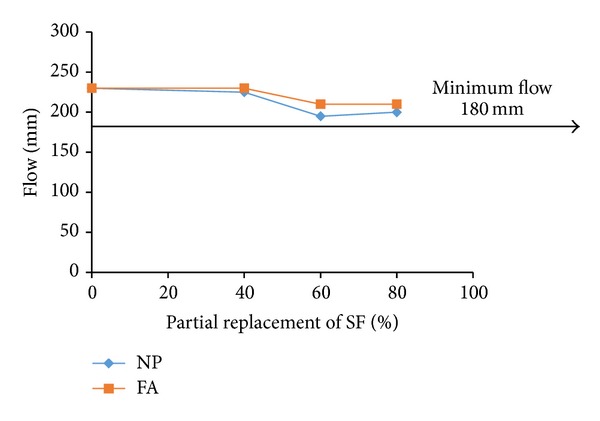
Variation of flow with replacement of SF by NP and FA.

**Figure 4:**
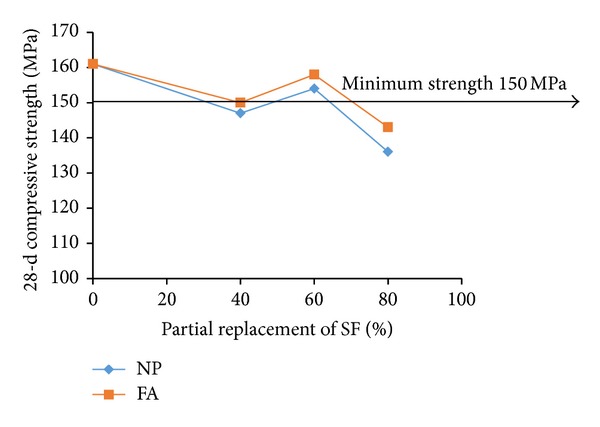
Variation of compressive strength with replacement of SF by NP and FA.

**Figure 5:**
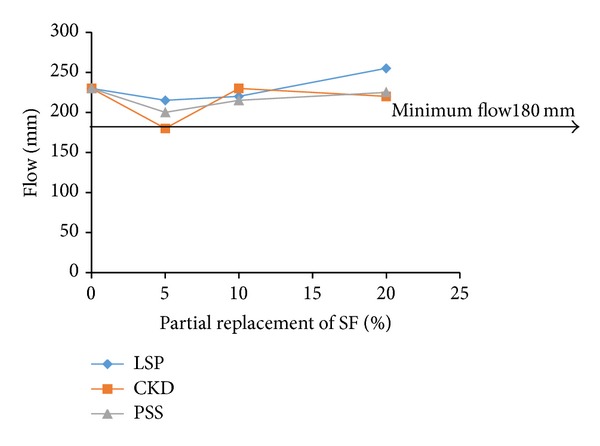
Variation of flow with replacement of SF by LSP, CKD, and PSS.

**Figure 6:**
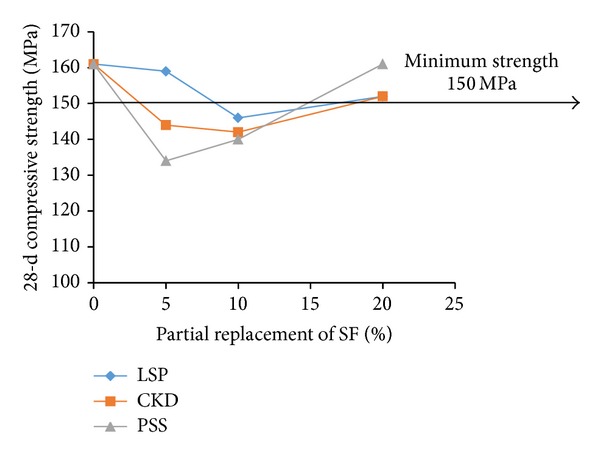
Variation of compressive strength with replacement of SF by LSP, CKD, and PSS.

**Figure 7:**
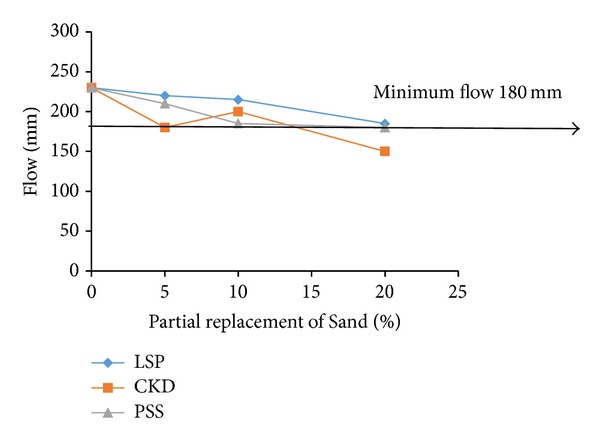
Variation of flow with replacement of sand by LSP, CKD, and PSS.

**Figure 8:**
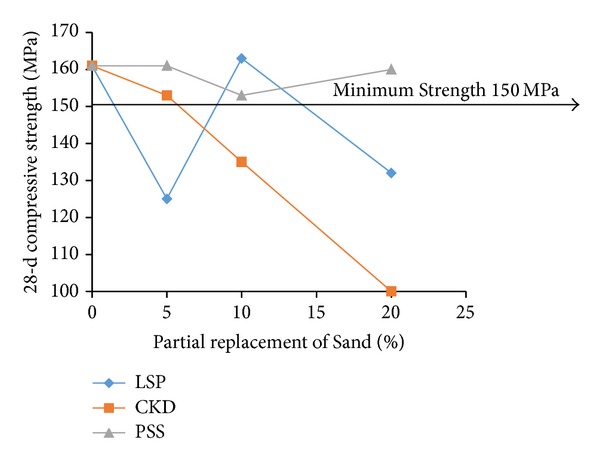
Variation of compressive strength with replacement of sand by LSP, CKD, and PSS.

For examining the acceptable levels of partial replacements of microsilica and dune sand by the replacing materials, the flow and 28-d compressive strength test results were plotted as shown in Figures 3, 4, 5, 6, 7, and 8. The minimum acceptable values of flow and 28-d compressive strength were considered as 180 mm and 150 MPa, respectively.

The plots of flow values obtained for UHPC mixtures with NP and FA replacing microsilica, as shown in Figure 3, indicate that the flow is more than minimum limit at all levels of replacement. However, as can be seen from Figure 4, it is found that the minimum required 28-day strength can be achieved at an optimum replacement level of 60% for both NP and FA. Therefore, it can be concluded that the optimum dosages of NP and FA for partially replacing the microsilica are typically found to be 60%. Like the case of mixtures with NP and FA, plots shown in Figure 5 indicate that the UHPC mixtures with LSP, CKD, and PSS, partially replacing the microsilica, can achieve minimum required flow at all levels of replacements. However, the minimum required 28-d compressive strength of 150 MPa can be obtained only at a replacement level of 20% for each of the three replacing materials (LSP, CKD, and PSS). Thus, the optimum level of partial replacement of microsilica by LSP, CKD, and PSS is typically 20%.

Referring to Figures 7 and 8 for examining the optimum levels of partial replacement of dune sand by LSP, CKD, and PSS, it is observed that all three levels of partial replacements of dune sand by PSS are acceptable because minimum required flow and strength are satisfied with all three replacement levels for PSS. All three levels of replacement by LSP also satisfy the minimum required flow but the minimum 28-d compressive strength is achievable only at 10% of replacement of dune sand by LSP. Although the minimum flow was satisfied when dune sand was replaced by CKD at 5% and 10% levels, the minimum required 28-d compressive strength was achieved with 5% CKD. Therefore, it can be concluded that while the optimum levels of replacement of dune sand by CKD and LSP were typically 5% and 10%, respectively, the PSS can replace dune sand by 5%, 10%, and 20% without compromising with the minimum required flow and 28-d compressive strength.

The finally selected 10 UPHC mixtures meeting the criteria for minimum required flow (180 mm) and 28-d compressive strength (150 MPa) are listed in Table 9. The flexural tensile strength measured for these mixtures was also determined as shown in Table 9. The flexural tensile strength of the developed UHPC mixtures is found to vary in the range of 23 to 32 MPa, which is about two to three times more than the traditional concrete having similar strength grade. This increase in the tensile strength is attributed to the presence of steel fibers added to the UHPC mixture.

## 4. Conclusions

Based on the findings of the present study, the following conclusions can be drawn.

- The possibility of utilizing the natural and industrial waste materials considered in this study for development of UHPC mixtures as a partial replacement of microsilica as well as sand is confirmed. The outcomes of this study would be beneficial particularly in reducing the consumption of microsilica, which is relatively a costly material in producing UHPC in Saudi Arabia.
- The optimum level of replacing microsilica by NP and FA was typically found to be 60%, whereas it was 20% in case of LSP, CKD, and PSS.
- While the optimum levels of replacing dune sand by CKD and LSP were 5% and 10%, respectively, the PSS can be used to replace the dune sand at all three levels, 5%, 10%, and 20%, without compromising with the minimum required flow and 28-d compressive strength.
- As listed in Table 8, a total of 10 mixtures of UHPC were developed utilizing natural and industrial waste materials as partial replacements of silica fume and dune sand. Apart from high compressive strength, these mixtures have about two to three times more flexural tensile strength as compared to traditional concrete with similar strength grade.

## Figures and Tables

**Table 1:** Chemical composition of cement.

Constituent	Weight %
CaO	64.35
SiO_2_	22.0
Al_2_O_3_	5.64
Fe_2_O_3_	3.80
K_2_O	0.36
MgO	2.11
Na_2_O	0.19
Equivalent alkalis (Na_2_O + 0.658K_2_O)	0.33
SO_3_	2.10
Loss on ignition	0.7

**Table 2:** Grading of the dune sand used as aggregate.

ASTM sieve number	Size (mm)	Percentage passing (%)
4	4.75	100
8	2.36	100
16	1.18	100
30	0.6	75
50	0.3	10
100	0.15	5

**Table 3:** Technical data of Glenium 51 used as superplasticizer.

Item	Description
Appearance	Brown liquid
Specific gravity at 20°C	1.08 ± 0.02 g/cm^3^
pH-value at 20°C	7.0 ± 1.0
Alkali content	≤5.0
Chloride content	≤0.1%

**Table 4:** Chemical composition of the microsilica.

Constituent	Weight %
SiO_2_	92.5
Al_2_O_3_	0.72
Fe_2_O_3_	0.96
CaO	0.48
MgO	1.78
K_2_O	0.84
Na_2_O	0.5
Loss on ignition	1.55

**Table 5:** Details of the materials used as partial replacements of microsilica and sand.

Replacing materials	Source	Specific gravity	CaO (% by mass)	SiO_2_(% by mass)
Natural pozzolana (NP)	Volcanic rocks in Western Province of Saudi Arabia	3.00	8.06	42.13
Fly ash (FA)	Local ready mixed concrete company in Saudi Arabia	2.25	8.38	45.30
Lime stone powder (LSP)	Local aggregate quarry in Abu Hadriyah, Saudi Arabia	2.60	45.70	11.79
Cement kiln dust (CKD)	Saudi Cement Company, Jeddah, Saudi Arabia	2.79	49.30	17.10
Pulverized steel slag (PSS)	Local steel manufacturing company in Saudi Arabia	3.75	40.80	16.47

**Table 6:** Quantities of constituents for producing 1 m^3^ of the base UHPC mixture.

Cement kg	Fine dune sand kg	Water kg	Microsilica (MS) kg	Steel fibers kg	Plasticizer Glenium 51 kg
900	1005	163	220	157	40

**Table 7:** Quantities of all ingredients for producing 1 m^3^ of the trial UHPC mixtures.

Partial replacement of microsilica and sand	Mixture ID	Cement kg	Sand kg	Water kg	Microsilica (MS) kg	Replacing material kg	Steel fibers kg	Plasticizer Glenium 51 kg
Base mixture without replacement	BMWR	900	1005	162	220	0	157	40

Natural pozzolana (NP) replacing 40%, 60%, and 80% microsilica (MS)	NP40RMS	900	1030	162	132	88	157	40
	NP60RMS	900	1042	162	88	132	157	40
	NP80RMS	900	1055	162	44	176	157	40

Fly ash (FA) replacing 40%, 60%, and 80% microsilica (MS)	FA40RMS	900	1005	162	132	88	157	40
	FA60RMS	900	1005	162	88	132	157	40
	FA80RMS	900	1005	162	44	176	157	40

Lime stone powder (LSP) replacing 5%, 10%, and 20% microsilica (MS)	LSP05RMS	900	1000	162	209	11	157	40
	LSP10RMS	900	995	162	198	22	157	40
	LSP20RMS	900	985	162	176	44	157	40

Cement kiln dust (CKD) replacing 5%, 10%, and 20% microsilica (MS)	CKD05RMS	900	1008	162	209	11	157	40
	CKD10RMS	900	1010	162	198	22	157	40
	CKD20RMS	900	1015	162	176	44	157	40

Pulverized steel slag (PSS) replacing 5%, 10%, and 20% microsilica (MS)	PSS05RMS	900	1010	162	209	11	157	40
	PSS10RMS	900	1015	162	198	22	157	40
	PSS20RMS	900	1025	162	176	44	157	40

Lime stone powder (LSP) replacing 5%, 10%, and 20% sand	LSP05RSAND	900	931	162	220	47	157	40
	LSP10RSAND	900	868	162	220	87	157	40
	LSP20RSAND	900	764	162	220	153	157	40

Cement kiln dust (CKD) replacing 5%, 10%, and 20% sand	CKD05RSAND	900	962	162	220	48	157	40
	CKD10RSAND	900	922	162	220	92	157	40
	CKD20RSAND	900	851	162	220	170	157	40

Pulverized steel slag (PSS) replacing 5%, 10%, and 20% sand	PSS05RSAND	900	972	162	220	49	157	40
	PSS10RSAND	900	942	162	220	94	157	40
	PSS20RSAND	900	886	162	220	177	157	40

**Table 8:** Flow and compressive strength test results.

Mixture ID	Flow (mm)	28-d compressive strength (MPa)	Mixture ID	Flow (mm)	28-d compressive strength (MPa)
BMWR	230	161	LSP05RSAND	220	125
NP40RMS	225	147	LSP10RSAND	215	163
NP60RMS	195	154	LSP20RSAND	185	132
NP80RMS	200	136	CKD05RSAND	180	153
FA40RMS	230	150	CKD10RSAND	200	135
FA60RMS	210	158	CKD20RSAND	150	100
FA80RMS	210	143	PSS05RSAND	210	161
LSP05RMS	215	159	PSS10RSAND	185	153
LSP10RMS	220	146	PSS20RSAND	180	160
LSP20RMS	255	152			
CKD05RMS	180	144			
CKD10RMS	230	142			
CKD20RMS	220	152			
PSS05RMS	200	134			
PSS10RMS	215	140			
PSS20RMS	225	161			

**Table 9:** Selected UPHC mixtures meeting the criteria for minimum required flow (180 mm) and 28-d compressive strength (150 MPa).

Mix ID	Flow (mm)	28-d compressive strength (MPa)	28-d flexural tensile strength (MPa)
BMWR	230	161	31
NP60RMS	195	154	29
FA60RMS	210	158	32
LSP20RMS	255	152	31
CKD20RMS	220	152	25
PSS20RMS	225	161	25
LSP10RSAND	215	163	29
CKD05RSAND	180	153	26
PSS05RSAND	210	161	24
PSS10RSAND	185	153	23
PSS20RSAND	180	160	24
